# Exploration of different quantitative polymerase chain reaction-based genotyping methods to distinguish *Apc*^min/+^ mice from wildtype mice

**DOI:** 10.1371/journal.pone.0317038

**Published:** 2025-02-04

**Authors:** Yuting Sun, Tingyu Zhou, Silin Ye, Effie Yin Tung Lau, Yao Zeng, Jessie Qiaoyi Liang

**Affiliations:** Department of Medicine and Therapeutics, Li Ka Shing Institute of Health Sciences, The Chinese University of Hong Kong, Hong Kong, China; Cornell University, UNITED STATES OF AMERICA

## Abstract

Various molecular methods have been established for genotyping single-nucleotide variants (SNVs). However, despite the widespread availability of quantitative polymerase chain reaction (qPCR) instruments in biomedical laboratories, the lack of professional analytical tools impedes the application of qPCR in genotyping. *Apc*^min/+^ mice, which harbour a germline *Apc* mutation (g.2549T>A) associated with multiple intestinal neoplasms, are extensively employed in colorectal cancer research. In this study, we used *Apc* as a model and assessed the feasibility of different qPCR-based methods for SNV genotyping, considering approaches with and without genotyping analytical tools. We initially employed allele-specific PCR followed by electrophoresis to determine the genotypes of *Apc* in tail tissues from potential *Apc*^min/+^ mice, and this method served as the benchmark for evaluating the performance of qPCR-based methods. Dye-based qPCR and melting curve assays exhibited distinct dissociation patterns that differentiated between synthesised wildtype (TT) and heterozygous mutant (TA) DNA and between TT and TA genotype mice based on analysis of tissue samples. This discrimination ability of these assays was unaffected by the use of different intercalating dyes (SYBR Green I or EvaGreen). Dual-probe qPCR assays were developed to simultaneously detect mutant and wildtype alleles using differently labelled probes. The genotyping module and delta cycle threshold method were used to facilitate the analysis of results. The qPCR-based methods displayed 100% agreement with the standard genotyping outcomes. When the PCR–electrophoresis method was used, approximately 15% of the samples required re-examination to obtain conclusive results. In contrast, when the qPCR methods were used, success rates exceeding 99% were achieved with a single test. Additionally, all qPCR-based methods determined mouse genotypes by analysis of stool samples, highlighting the applicability of these methods for non-invasive genotyping. Loss of heterozygosity in the *Apc* gene in intestinal polyps was detected using the dual-probe assay with delta cycle threshold method. In summary, this study successfully implemented intercalator-based and probe-based qPCR methods, with and without professional analytical modules, for characterising *Apc* in tissue and stool samples. Furthermore, these methods can be extended to allow genotyping of other SNVs and can facilitate non-invasive genotyping of transgenic animals.

## Introduction

Single-nucleotide variant (SNV) genotyping methods play a crucial role in various fields, such as research, medicine, agriculture, forestry, and husbandry, in which they are used to identify single-nucleotide polymorphisms (SNPs) in plant and animal species. Typically, these methods employ polymerase chain reaction (PCR), microarrays, and sequencing techniques to detect SNPs, which are single-base substitutions.

The human adenomatous polyposis coli gene (*APC*) encodes a 310-kDa protein with β-catenin-binding sites [1] and is considered to be a tumour suppressor gene. Mutations in human *APC* lead to the spontaneous development of intestinal adenomas and are prevalent in approximately 80% of human colon tumours [2,3]. The *Apc*^min/+^ mouse model carries a heterozygous nonsense mutation (g.2549T>A, p.850L>X) in mouse *Apc*, the homologue of human *APC*. Consequently, this model is highly susceptible to developing spontaneous intestinal adenomas at 3–4 months of age and thus is extensively utilised in colorectal cancer research [4,5].

Several methods have been reported for genotyping mice to determine whether they harbour wildtype *Apc* or heterozygous mutant *Apc*. In 1993, Dietrich *et al* introduced an allele-specific PCR (AS-PCR) for the detection of the mutant allele [6]. This method involves targeted PCR amplification using common primers for both mutant and wildtype alleles, along with a specific primer for the mutant allele. Genotypes are determined by gel electrophoresis, which generates one band for wildtype samples and two bands for *Apc*^*min/+*^ samples. The PCR–restriction fragment length polymorphism (PCR–RFLP) method was developed by Luongo et al. in 1994 [2]. This method involves PCR amplification of the target region, enzymatic digestion of PCR products, and gel electrophoresis of the digested PCR products for genotype analysis. In 2012, Symonds and Fenech incorporated AS-PCR and dissociation curve analysis on a real-time PCR machine, eliminating the need for gel electrophoresis [7]. However, this technique generates PCR products of considerable sizes, including a mutant allele-specific PCR product of 331 bp and another PCR product common to both wildtype and mutant alleles at 619 bp. These sizes could potentially impact the testing success rate on DNA samples of limited quality and quantity, such as fecal DNA samples containing a small proportion of host DNA. Recent advances in genotyping techniques have led to the introduction of new methods, such as allele-specific hybridisation chain reaction, which incorporates isothermal amplification and probe hybridisation in an array format [8]. Additionally, clustered regularly interspaced short palindromic repeats (CRISPR)–CRISPR-associated protein 12 (Cas12) assays have been developed that exploit the kinetic shifts in Cas12 trans-cleavage substrate affinity and catalytic efficiency between mutants and wildtypes [9]. However, these novel methods may require specialised equipment and expertise, limiting their widespread adoption.

qPCR is a widely used technique in molecular biology, and most biomedical researchers are familiar with its principles and applications. Two commonly used qPCR-based methods are intercalator-based high-resolution melting (HRM) analysis and probe-based genotyping. Moreover, qPCR instruments are ubiquitous in general molecular biology laboratories. Therefore, qPCR-based methods are the most convenient and accessible options for SNV genotyping, making them highly suitable for routine genotyping tasks.

The T>A mutation in *Apc*^*min/+*^ mice is a member of the most difficult type of SNVs (class 4) to discriminate, especially by the HRM method. Accordingly, in this study, we focused on the T>A mutation of *Apc* (g.2549T>A) and investigated various qPCR-based methods for genotyping mice to determine whether they bore this mutation. We also explored alternative analytical methods based on melting behaviours and delta cycle threshold **(Δ**Ct) values, which do not require specialised genotyping tools. Additionally, we evaluated the utility of qPCR-based methods for non-invasive genotyping of mice by analysis of stool samples.

## Materials and methods

### Tissue biopsies and stool samples from mice

As part of the standard procedure for breeding C57BL/6J-*Apc*^*min/+*^ mice, pinna biopsies were collected by trained personnel from the Laboratory Animal Services Centre at The Chinese University of Hong Kong. Ear punches were conducted when the mice reached two weeks of age or when the pinna was fully developed. A 2-mm notch was excised from the pinna to obtain sufficient DNA for genotyping. Throughout the process, the ear punch apparatus was kept sterile and disinfected. A total of 145 ear biopsies were obtained. In the development of qPCR-based methods, 42 tissue DNA samples (20 wildtype and 22 *Apc*^*min/+*^) were utilised. For validation tests, 103 tissue DNA samples (49 wildtype and 54 *Apc*^*min/+*^) were employed. Stool collection involved placing each mouse in a sterile box for less than 30 min to obtain fresh stool samples. Stool collection was conducted at 3~4 weeks of age following confirmation of genotypes using tissue biopsies. Stool samples were acquired from 80 mice, including 38 wildtype and 42 *Apc*^*min/+*^. These stool samples were solely used to assess the feasibility of our methods in non-invasive genotyping; hence, a smaller sample size was involved. The mice’s well-being was monitored daily, and their body weights were recorded weekly. This study was approved by the Animal Ethics Committee of The Chinese University of Hong Kong (AEEC#: 21-107-NSF, approval date: March 17, 2021). Following the completion of this study, all mice transitioned into other research projects as per the protocols set forth by our university’s animal center and in accordance with the ethical approvals by the Animal Ethics Committee of The Chinese University of Hong Kong for each respective study. Mice were euthanized by anesthetic carbon dioxide gas at the end of experiments. Following euthanasia, non-polyp colon tissues (n = 22) and polyps (n = 10, not pure for small polyps) were collected from 12-week-old *Apc*^*min/+*^ mice.

### Tissue samples and DNA extraction

Each tissue sample was immediately placed in a 1.5-mL tube in liquid nitrogen, which was then stored at -80°C until DNA extraction. Lysis buffer was prepared and used for DNA extraction, as previously reported [10]. Briefly, 200 μL of lysis buffer was added to 0.1 mg of each sample, and the resulting mixture was incubated at 55°C overnight. Subsequently, DNA was precipitated by isopropanol treatment, collected, washed with ice-cold 70% ethanol, dried, and finally dissolved in 50 μL of ultrapure water. The resulting DNA samples were stored at -80°C until further use.

### Stool samples and DNA extraction

Stool samples were stored at -80°C until DNA extraction. DNA was extracted from a pellet of stool from each mouse using a High-yield Magnetic Stool Feces DNA Extraction Kit (BayBio Bio-tech, Guangzhou, China), according to the manufacturer’s instructions for stool and host DNA extraction. Briefly, a suspension buffer, lysis buffer, and proteinase K were added to a sample, and the resulting mixture was thoroughly vortexed and then incubated at 70°C for 20 minutes. After centrifugation, the supernatant was transferred into a new tube for DNA purification using magnetic beads. DNA samples were stored in -80°C for further use.

### AS-PCR followed by gel electrophoresis to detect mutant alleles

The nucleotide sequences of the primers used were described previously [7] and are listed in **Table 1**. Each PCR reaction contained 10 μL of 2× PCR premix (Premix Taq™ DNA Polymerase Hot-Start Version, TaKaRa Bio Inc, Dalian, China), 4–6 pmol of each primer, 10 ng of template DNA, and 20 μL of ultrapure water. A negative PCR control containing all reagents except template DNA was prepared for each experiment. PCR amplifications were performed on a ProFlex PCR System (Applied Biosystems, Waltham, MA) using the following cycling parameters: 95°C for 5 minutes; followed by 35 cycles of 95°C for 15 seconds, 55°C for 15 seconds, and 72°C for 1 minute; and then final extension at 72°C for 1 minute. Five microliters of PCR products were separated by gel electrophoresis on 2% (w/v) agarose gel, and gel red was used for staining. A 619-bp common band was exhibited by wildtype and mutant alleles, whereas a 331-bp band was only exhibited by the mutant allele. These genotyping results were used as standard results, and the genotyping of randomly selected samples was further confirmed by Sanger sequencing.

**Table 1:** Nucleotide sequences of primers and probes used in this study.

Primer/probe	Sequence (5’—>3’)	Description
**Apc-cF**	TTCCACTTTGGCATAAGGC	For AS-PCR*
**Apc-cR**	GCCATCCCTTCACGTTAG	
**Apc-mR**	TTCTGAGAAAGACAGAAGTTA	
**Apc-F**	GGTATTGCCCAGCTCTTCTT	For dual-probe qPCR
**Apc-wt-FAM**	5’Fam-TTGGAGAGAGAGCGAGGTATTGGCC-3’TAMRA	
**Apc-mut-VIC**	5’VIC-TAGGAGAGAGAGCGAGGTATTGGCC-3’TAMRA	
**Apc-R**	GTTGTTGGATGGTAAGCACTG	
**Apc-F**	GGTATTGCCCAGCTCTTCTT	For dye-based qPCR
**Apc-R**	GTTGTTGGATGGTAAGCACTG	

*primers as described by Symonds and Fenech [7].

### qPCR

All intercalator dye-based or probe-based qPCR amplifications were performed in MicroAmp Fast Optical 96-well reaction plates with adhesive sealing on an ABI QuantStudio 7 Flex Real-Time PCR System (Applied Biosystem). Duplicate analyses of each sample were performed. A positive control, or standard controls, and a negative control (with water as a template) were included in every experiment. qPCR data was analysed using Design & Analysis Software 2.6.0 (Applied Biosystems) with manual settings, i.e., a threshold of 0.05 and a baseline obtained from 3–15 cycles for all samples. Experiments were disqualified if their negative control Ct values were less than 38, and samples were disqualified if their Ct values were greater than 36. Both intercalator dye-based and probe-based qPCR assays utilised the same pair of primers (**Table 1**). The specificity of PCR amplification and genotyping accuracy were confirmed through direct Sanger sequencing of randomly selected PCR products.

### Sanger sequencing

Direct Sanger sequencing was conducted using the Apc-F and Apc-R primers (**Table 1**) on an ABI 3730XL system (Applied Biosystems). The results were analysed using Chromas 2.6.6 (Technelysium Pty Ltd, South Brisbane, Australia).

### SYBR Green I-based qPCR and melting curve assay

We used TB Green (SYBR Green I) Premix Ex Taq™ (Tli RNaseH Plus) (TaKaRa), which is commonly used in our laboratory for intercalator-based real-time PCR. Each qPCR reaction contained 10 μL of 2× PCR Premix, 0.4 μL of ROX ІІ, 10 ng of a DNA sample, 4 pmol of each primer, and ultrapure water to 20 μL. The cycling and melting conditions were as follows: 95°C for 30 seconds, 40 cycles of 95°C for 3 seconds and 60°C for 30 seconds, followed by 95°C for 15 seconds, 60°C for 1 minute, an increase to 95°C at 0.05°C/second with signals captured continuously, and then 95°C for 15 seconds. The nucleotide sequences of primers are listed in **Table 1**.

### EvaGreen-based qPCR and melting curve assay

A Type-it HRM PCR Kit (Qiagen, Hilden, Germany) was used, as it contains the saturating DNA binding dye EvaGreen and is optimised for HRM analysis. Each qPCR reaction contained 10 μL of 2× Type-it HRM PCR Master Mix, 4 pmol of each primer, 10 ng of a DNA sample, and ultrapure water to 20 μL. The thermal cycler parameters were as follows: 95°C for 5 minutes, 40 cycles of 95°C for 10 seconds and 55°C for 30 seconds, followed by 95°C for 15 seconds, 60°C for 1 minute, an increase to 95°C at 0.025°C/second with signals captured continuously, and 95°C for 15 seconds.

### TaqMan dual probe-based SNP assay using a genotyping analysis module

qPCR reactions contained two primers and two differentially labelled probes, as listed in **Table 1**. Each probe carried a 5′ reporter dye (6-carboxy fluorescein or 4,7,2′-trichloro-7′-phenyl-6-carboxyfluorescein) and a 3′ quencher dye (6-carboxytetramethyl-rhodamine). Hydrolysis probes were obtained from Invitrogen (Carlsbad, CA). PCR reactions were performed in 20-μL reactions containing 10 μL of 2× Premix Ex Taq master mix for probe-based real-time PCR (TaKaRa), 0.2 μL of ROX ІІ (contained in the kit), 10 ng of a DNA sample, 5 pmol of each primer, and 3 pmol of each probe. The cycling parameters were as follows: 95°C for 30 seconds, followed by 40 cycles of 95°C for 5 seconds and 60°C for 30 seconds, and finally 60°C for 30 seconds for the post-read stage.

### Dual probe-based ΔCt assay

The PCR reagents and setups were the same as for the SNP assay using a genotyping module. The cycling parameters were as follows: 95°C for 30 seconds, followed by 40 cycles of 95°C for 5 seconds and 60°C for 30 seconds. Data analysis was carried out according to the ΔCt method, with ΔCt = Ct_mut_ − Ct_wt_, and type score = Power (2, -ΔCt).

### Statistical analysis

Data are reported as means ± standard deviations (SDs) or medians (interquartile ranges [IQRs]), as appropriate. Differences in scores were determined using an ordinary one-way ANOVA test followed by Tukey’s multiple comparisons. Receiver operating characteristic curves were used to evaluate the diagnostic value of measurements. All tests were conducted using GraphPad Prism 9.4 (GraphPad Software Inc., San Diego, CA). A *P* value < 0.05 was regarded as indicating a statistically significant difference.

## Results

### DNA samples

The DNA samples extracted from tail tissues of mice exhibited a 260-to-280-nm optical dispersion (OD260/280) ratio of 2.36 ± 0.87 (mean ± SD) and a 260-to-230-nm optical dispersion (OD260/230) ratio of 1.65 ± 0.31. In contrast, the DNA samples extracted from the stool samples of potential mutant mice had an OD260/280 ratio of 2.05 ± 0.07 and an OD260/230 ratio of 1.50 ± 0.57. For PCR/qPCR, we utilised 10 ng of tissue or stool DNA, or 2–5 μL directly if the DNA concentration was too low. In probe-based qPCR assays of tissue samples, the median Ct values for the mutant and wildtype alleles were 27.6 (IQR: 27.1–27.9) and 27.2 (26.7–27.6), respectively. For such assays of stool samples, the Ct values were 33.4 (32.2–34.1) and 33.2 (32.0–34.2) for the mutant and wildtype alleles, respectively. In SYBR Green I-based qPCR assays, the Ct values were 21.7 (21.4–21.9) for tissue samples and 26.8 (26.1–27.8) for stool samples. These differences in Ct values between tissue and stool samples were expected, as stool DNAs are of lower quality than tissue DNAs and are predominantly composed of microbial DNA and limited mouse DNA.

### Genotyping by AS-PCR followed by gel electrophoresis

**Fig 1:**
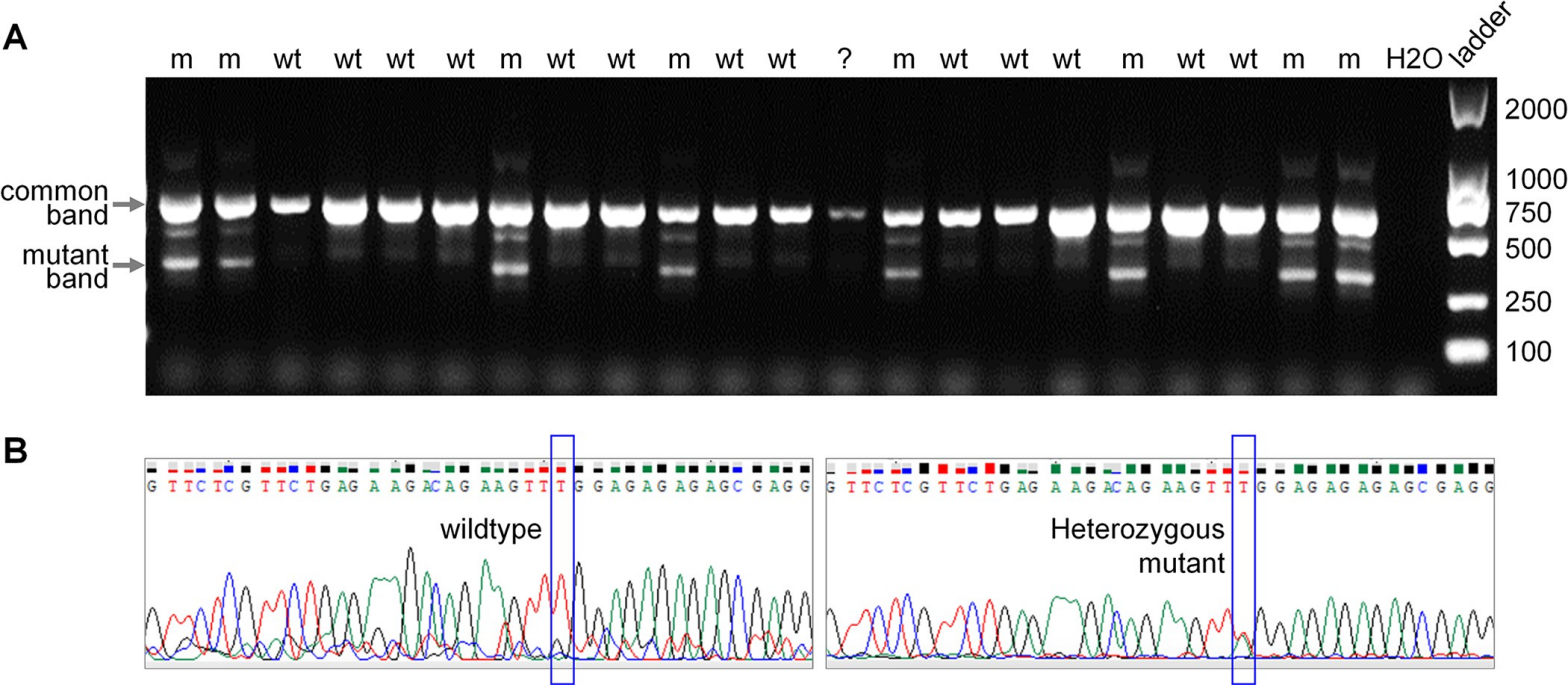
(**A**) Gel electrophoresis results of allele-specific PCR (AS-PCR) conducted on tail tissues obtained from potential breeders of Apc^min/+^ mice. Heterozygous mutants (m) were identified based on the presence of both a 619-bp common band and a 331-bp mutant-specific band. Wildtypes (wt) were identified by the presence of only the 619-bp common band. Samples marked with ‘?’ were undetermined and necessitated further examination. (**B**) Representative chromatograms of PCR products from wildtype and *Apc*^Min/+^ mice by Sanger sequencing.

We employed AS-PCR, which was developed by Symonds and Fenech [7], to genotype all samples. Gel electrophoresis was performed to visualise the genotypes, as shown in **Fig 1A**. Wildtype samples exhibited a single band, while heterozygous mutants displayed two bands. These genotyping results served as the standard for evaluating the accuracy of the newly established methods, with genotyping of randomly selected samples also confirmed by Sanger sequencing (**Fig 1B**). Approximately 15% of the tissue DNA samples required re-examination to obtain conclusive genotyping results, due to problems such as weak PCR bands and ambiguous mutant bands.

### Genotyping by dye-based qPCR and melting curve analysis

**Fig 2:**
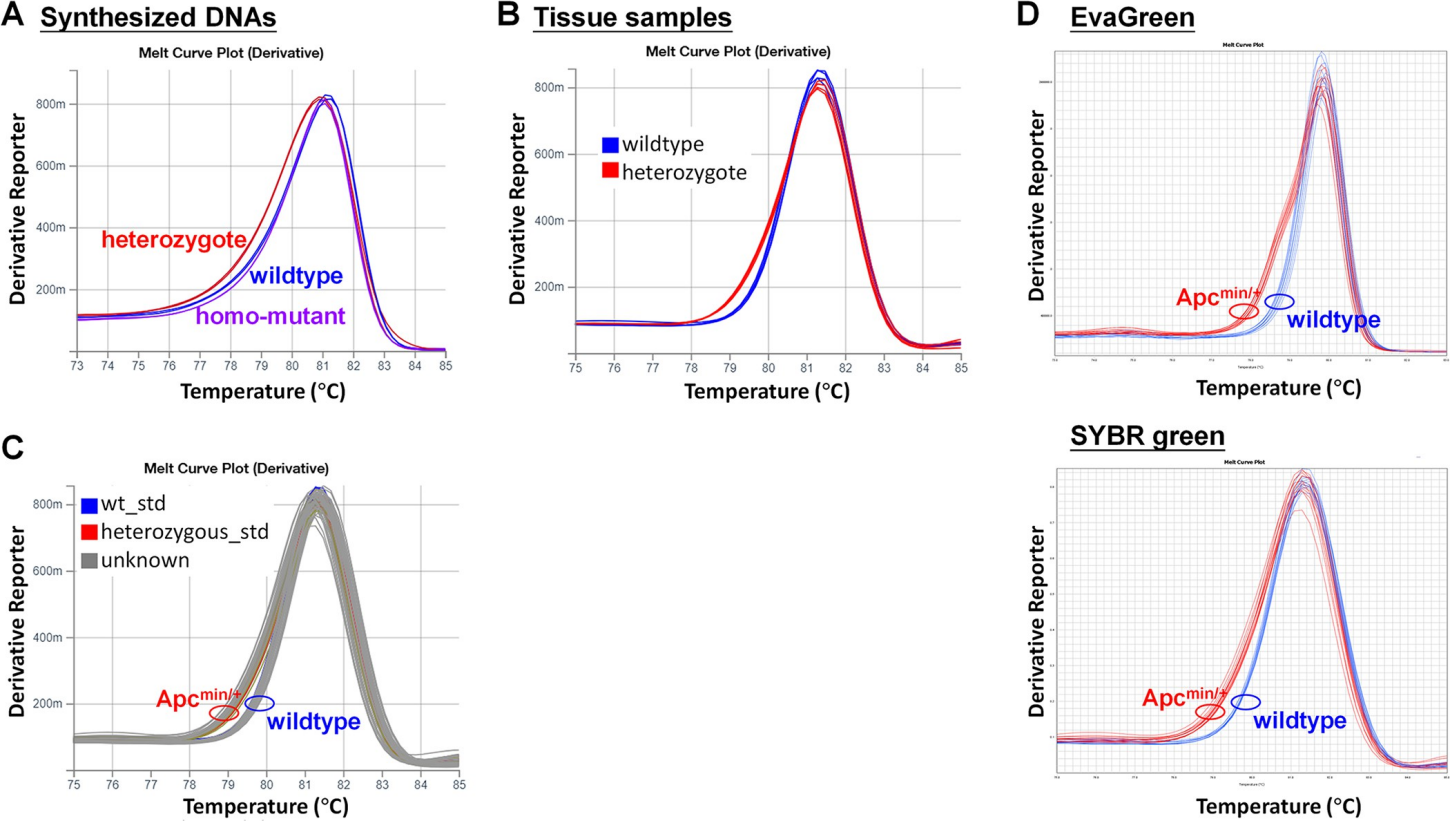
Genotyping using dye-based qPCR assays. (**A**) Melting curves of synthesized homozygous mutant, wildtype, and heterozygous mutant targets. (**B**) Melting curves of tissue samples with known genotypes. (**C**) Melting curves of wildtype and heterozygous mutant standards, along with some testing samples. (**D**) Melting curves of tissue samples with known genotypes obtained using EvaGreen and SYBR green assays.

A pair of primers was used to amplify a 114-bp region encompassing the mutation site, utilising the standard curve mode to detect intercalator signals, as in regular dye-based qPCR coupled with an additional melt-curve stage. Initially, we employed the widely used dye SYBR Green I. The melting curves of synthesised heterozygous mutant DNA exhibited distinguishable patterns compared with those of synthesised wildtype and homozygous mutant DNA. In contrast, the melting curves of synthesised wildtype and homozygous mutant DNA displayed similar curves, as expected (**Fig 2A**). The melting curves of tissue sample DNAs with known genotypes exhibited a clear separation between wildtypes and heterozygotes (**Fig 2B**). Hence, the genotypes of unknown samples could be classified by their proximity to the standard controls within the same experiment (**Fig 2C**). We also evaluated the utility of EvaGreen, which is a saturating DNA-binding dye that is expected to be more suitable for HRM analysis than SYBR Green I. The results showed that EvaGreen afforded distinguishable dissociation curves for wildtypes and heterozygous mutants, although the difference in dissociation modes between the two genotypes was not significantly greater than that obtained with SYBR Green I (**Fig 2D**). Consequently, we selected SYBR Green I for further testing due to its lower cost. A validation group of 103 samples was analysed using this classification method, and the results exhibited 100% concordance with results verified by PCR–electrophoresis, and no sample required re-examination for conclusive classification.

### TaqMan dual-probe-based SNP assay using a genotyping analysis module

**Fig 3:**
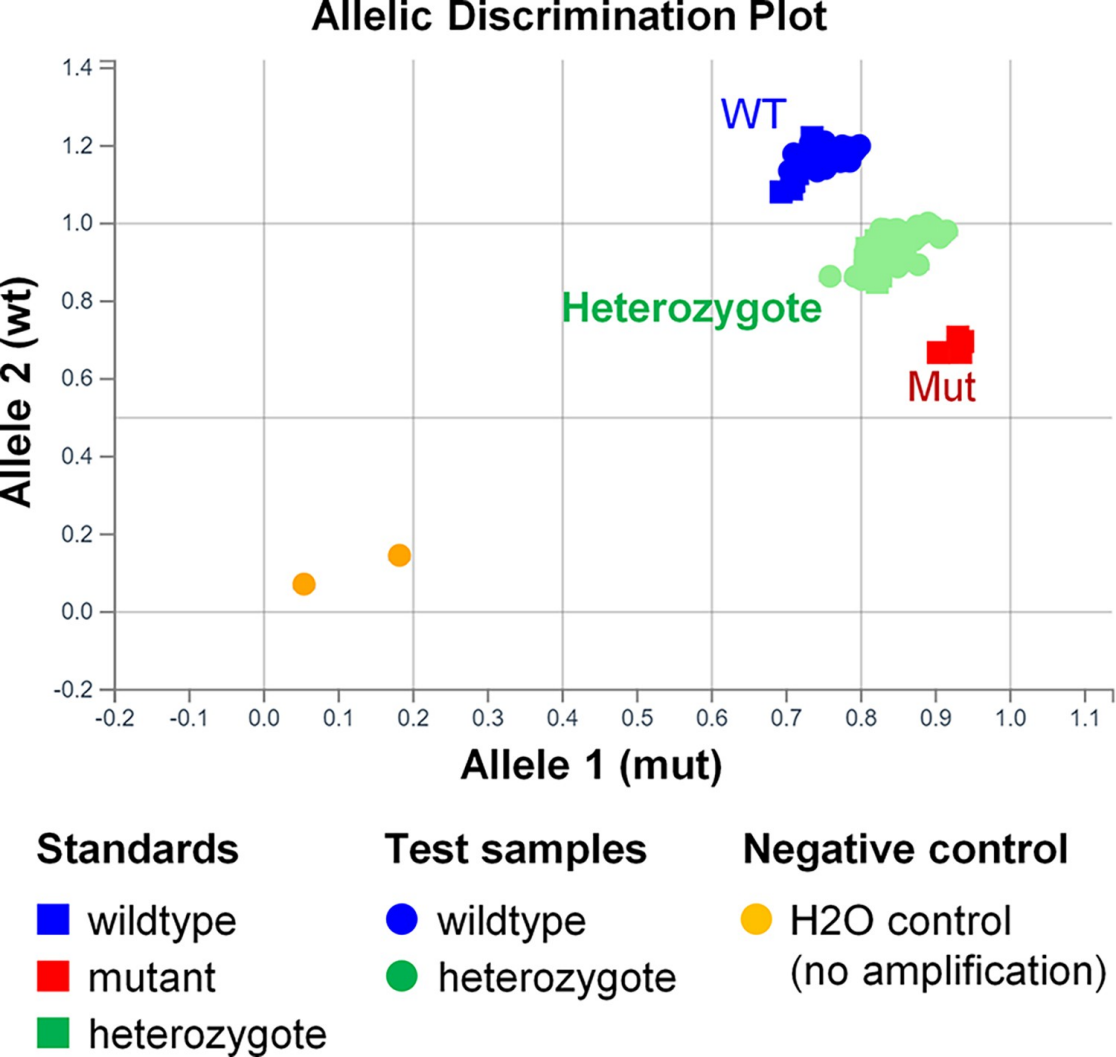
Genotyping using TaqMan dual-probe based SNP assay by genotyping module. Shown is an allelic discrimination plot displaying the distribution of various standard controls, including wildtype, heterozygous mutant, and homozygous mutant (synthesized) samples, as well as no template (H2O) control. Additionally, it shows the tested samples with their genotypes automatically called by the genotyping analysis module based on their fluorescence signals and their proximity to the standard controls.

To detect the wildtype and mutant alleles simultaneously, a pair of primers common to both alleles was designed, together with two TaqMan probes. One probe was specific for the wildtype allele, while the other was specific for the mutant allele, and each was functionalised with a different fluorescent label. By employing a genotyping analysis module, the genotypes of the tested samples could be automatically determined by comparing them with the synthesised standard controls, as shown in the allelic discrimination plot (**Fig 3**). This method is convenient, as it does not require additional user input to generate results. Moreover, the results of this method exhibited 100% concordance with the results verified by AS-PCR for both testing (*n* = 42) and validation (*n* = 103) groups, showcasing its accuracy and reliability.

### TaqMan dual-probe-based SNP assay using the ΔCt method

**Fig 4:**
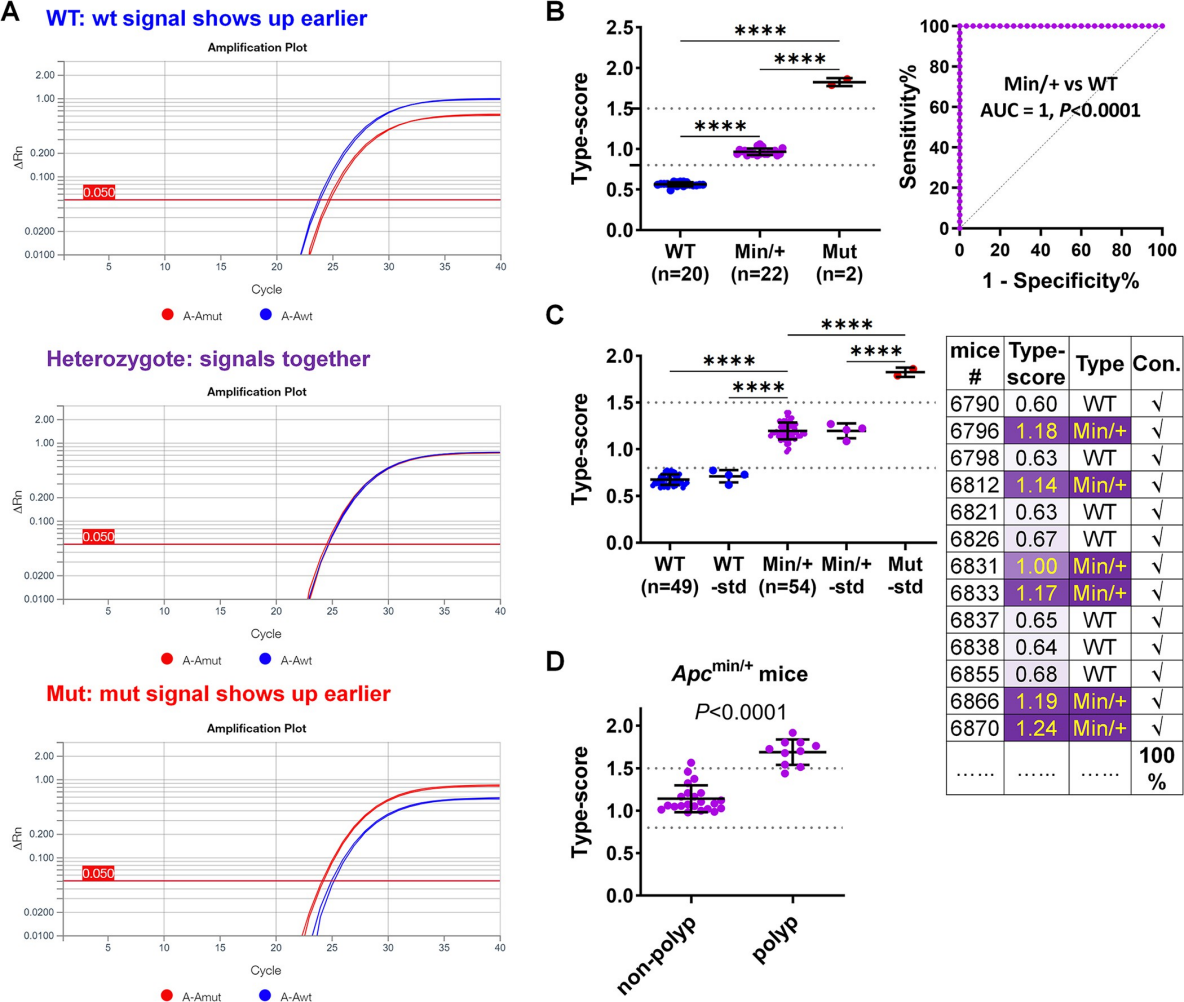
Genotyping using TaqMan dual-probe based SNP assay by ΔCt method. (**A**) Amplification plots illustrating the appearance of allele-specific signals in samples of different genotypes. WT, wildtype; Mut, homozygous mutant. (**B**) Type-scores in samples of three genotypes calculated using the ΔCt method and ROC curve discriminating heterozygous mutants (Min/+) from wildtypes (WT). (**C**) Type-scores of a validation group, with genotypes determined based on type-score thresholds: ≤0.8 for wildtype, 0.8–1.5 for heterozygotes and >1.5 for homozygous mutants. The predicted genotypes based on type-scores showed complete concordance with AS-PCR results. (**D**) Type-scores of non-polyp colon tissues (n = 22) and polyps (n = 10; not pure, containing adjacent tissues) from 12-week-old *Apc*^min/+^ mice. Data are mean ± SD. ****P<0.0001.

Without a genotyping analytic module on the qPCR equipment, we employed the ΔCt method to analyse the results of the TaqMan dual-probe SNP assay. The reagents and experimental procedure were the same as those in the above-described SNP assay using a genotyping module. The Ct values obtained by the set baseline and threshold were utilised for genotype analysis. In wildtype samples, the wildtype signal consistently appeared earlier than the mutant signal (Ct_wt < Ct_mut). In heterozygous samples, the wildtype and mutant signals appeared at similar times, resulting in similar Ct values. Conversely, in the synthesised homozygous mutant samples, the mutant signal emerged earlier than the wildtype signal (Ct_wt > Ct_mut) (**Fig 4A**). We therefore calculated the ΔCt to assess the difference between the three genotypes. The results demonstrated distinct and separate type-scores for each genotype: 0.565 ± 0.026 (mean ± SD) for wildtype samples, 0.966 ± 0.039 for heterozygote samples, and 1.825 ± 0.050 for homozygous mutant samples (**Fig 4B; S1 Table**). Consequently, we established cutoff values of 0.8 and 1.5 for classification purposes: ≤ 0.8 for wildtypes, 0.8–1.5 for heterozygotes, and > 1.5 for homozygous mutants. Samples in the validation group (*n* = 103) exhibited distinct type-scores, enabling unambiguous classification of samples as wildtypes or heterozygotes (**Fig 4C; S2 Table**). Only one sample out of 145 (0.7%) required re-analysis due to insufficient DNA template loading in one of the duplicate reactions. Notably, while the dye-based assay and dual-probe assay using a genotyping module were not sensitive to loss of heterozygosity, the type-scores of intestinal polyps from *Apc*^min/+^ mice significantly exceeded those of non-polyp tissues (**Fig 4D; S3 Table**), indicating the loss of the wildtype allele within the polyps.

### qPCR-based methods are efficient for non-invasive genotyping using stool samples

**Fig 5:**
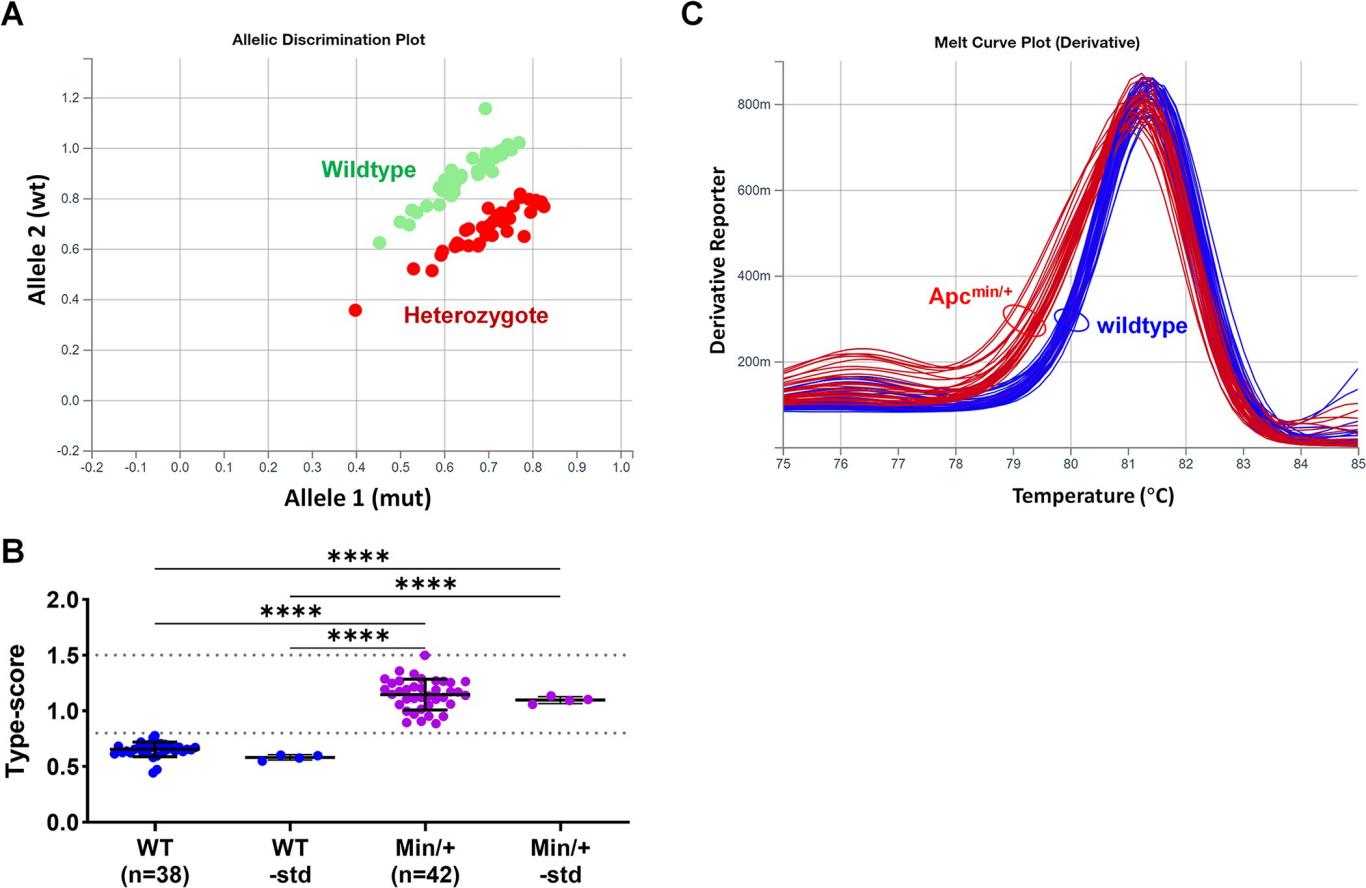
Genotyping stool samples using TaqMan dual-probe based SNP assays and dye-based qPCR assays. (**A**) Allelic discrimination plot correctly determined the genotypes of stool samples from mice of known genotypes, with standard controls of tissue samples included for reference. (**B**) Type-scores also correctly determined the genotypes of stool samples from mice of known genotypes. (**C**) Melting curves of stool samples of heterozygous mutants (*Apc*^Min/+^) and wildtypes exhibited clear separation, with dissociation modes resembling those of tissue samples. Data are mean ± SD. ****P<0.0001.

The efficiency of qPCR-based methods for genotyping using stool samples was evaluated. Stool samples were collected from mice with known forms of *Apc*, namely 38 wildtype mice and 42 *Apc*^*min/+*^ mice. Tissue DNA samples with known genotypes were used as standards during all qPCR assays. Both the dual-probe assay using the genotyping module and the ΔCt method successfully classified 96.3% (77/80) of the stool samples with 100% accuracy in a single test (**Fig 5A and 5B; S4 Table**). Additionally, the melt curve assay with SYBR Green I classified 93.4% (75/80) of the stool samples with 100% accuracy in a single test (**Fig 5C**). The unsuccessfully classified samples did not provide conclusive results due to factors such as low DNA quality or reagent preparation problems. However, upon re-examination (i.e., by increasing the DNA template amount or repeating the test), all of these samples were correctly genotyped. Overall, the qPCR-based methods demonstrated high accuracy and efficiency in genotyping stool samples, with a high percentage of correct classifications. The few unsuccessful samples were easily resolved through re-examination, ensuring accurate genotyping results.

## Discussion

In this study, we employed an *Apc* mouse model to investigate the feasibility of intercalator-based and probe-based qPCR methods, both with and without the utilization of professional analytical modules, for genotyping. These methods exhibited 100% accuracy when comparted to the standard AS-PCR method, offering additional advantages such as convenience, reduced requirements on DNA quality and quantity, higher success rates in testing, and the ease of obtaining conclusive results. Furthermore, we assessed the applicability of these methods for non-invasive genotyping through an analysis of stool samples. The findings illustrated that our assays were sensitive and effective for non-invasive genotyping. Furthermore, the techniques developed in this study can be easily extended to allow genotyping of other single-nucleotide substitution sites in various sample types.

qPCR is one of the most common molecular biology methods and can be widely used for genotyping due to its convenience and versatility. We demonstrated that genotyping can be accurately and efficiently performed using qPCR methods, even without professional analytical modules for genotyping. In our practical experience, compared with intercalator-based methods, dual probe-based methods offer greater convenience as they are less affected by sample quality, allowing for reliable comparisons across different sample types. A comparison of the advantages and disadvantages of different qPCR-based methods and the commonly used AS-PCR method is presented in **Table 2**. We note that when employing the melting curve method, it is important to use standard controls from the same sample type as the tested samples, as distinct dissociation behaviours are exhibited by different types of samples. For example, **Figs 2A, 2B and 5C** illustrate the distinct dissociation behaviours of synthesised DNAs, tissue samples, and stool samples in the temperature range of 75°C to 80°C. Nevertheless, the relative positions of wildtypes and heterozygous mutants remained consistent.

**Table 2:** The advantages and disadvantages of PCR/qPCR-based methods.

Method	Technical principle	Advantages	Disadvantages
AS-PCR	Electrophoresis to observe PCR products of different sizes representing different alleles	No need for qPCR instrument (conventional PCR machine is needed); lowest cost.	Tedious with gel electrophoresis; less sensitive; not closed-tube/need of handling PCR products may produce contamination.
Melting curve	Intercalator-based qPCR	Convenient; closed-tube; results easy to be interpreted.	Less sensitive for low-fraction mutations; not sensitive to discriminate between some homozygotes, such as those of class 4 SNVs; less stable and can be affected by template impurity, reagent contamination, etc.; standard controls must be used.
HRM	Intercalator-based qPCR	Convenient; closed-tube; results easy to be interpreted; sensitive for low-fraction mutations; no need for additional user input to achieve results.	Need professional analytical software; not sensitive to discriminate between some homozygotes, such as those of class 4 SNVs; standard controls must be used.
Dual-probe qPCR by genotyping module	Probe-based qPCR	Convenient; closed-tube; results easy to be interpreted; sensitive; no need for additional user input to achieve results.	Not cost-efficient if not used in large scale due to cost of probe synthesis; genotyping module is needed; standard controls must be used.
Dual-probe qPCR by ΔCt method	Probe-based qPCR	Convenient; closed-tube; results easy to be interpreted; sensitive; no need for standard controls (although qPCR controls are still needed).	Not cost-efficient if not used in large scale due to cost of probe synthesis; need post-qPCR data analysis to achieve results.

Conventional breeding programmes for research animal models often rely on invasive sampling methods, such as tail clipping, to obtain biopsies for genotyping. However, non-invasive methods based on stool samples were introduced in 1999 [11] and have been applied in various studies [7,12,13]. These non-invasive sampling methods not only improve animal welfare but are valuable for sampling endangered or elusive species and special transgenic animal models, including those with bleeding disorders.

In the current study, we successfully applied qPCR-based methods to genotype stool DNA samples extracted using a standard stool DNA extraction kit with proteinase K digestion. We focused on characterising *Apc* in the *Apc*^min/+^ mouse model, which is a common research model for colorectal cancer. We found that our qPCR-based methods, including those not requiring professional analytical modules, can readily replace conventional PCR-based methods. Furthermore, our qPCR methods were designed to detect the most challenging SNVs (class 4 SNVs) and thus can be easily extended to allow genotyping of other classes of SNVs and enable non-invasive genotyping of transgenic animals.

In conclusion, we successfully demonstrated the implementation of intercalator-based and probe-based qPCR methods, both with and without professional analytical modules, for characterising *Apc* in tissue and stool samples from mice. These novel intercalator-based and probe-based qPCR methods expand the application of qPCR-based genotyping and can also be easily extended to allow genotyping of other SNVs in various sample types.

## Supporting information

S1 Table Type-scores of Fig 4B. (XLSX)

S2 Table Type-scores of Fig 4C. (XLSX)

S3 Table Type-scores of Fig 4D. (XLSX)

S4 Table Type-scores of Fig 5B. (XLSX)
